# Promoting organizational vision integration among hospital employees

**DOI:** 10.1186/s12913-021-07430-z

**Published:** 2022-01-05

**Authors:** Terje Slåtten, Gudbrand Lien, Barbara Rebecca Mutonyi

**Affiliations:** Inland School of Business and Social Science, Inland Norway University of Applied Sciences, Campus Lillehammer, 2604 Lillehammer, Norway

**Keywords:** Employees, Organizational vision integration, Organizational commitment, Leadership autonomy support, Organizational culture, Hospital organizations

## Abstract

**Background:**

The concept of organizational vision has been little explored in the health-care services research literature. To address this knowledge gap in the literature, the present study examines the factors that may promote organizational vision integration (OVI), which refers to the employees’ use of organizational vision as a guiding framework in their work. The roles of organizational commitment (OC), leadership autonomy support (LAS), and organizational culture in relation to hospital employees’ OVI are examined.

**Methods:**

Hospital employees were surveyed. Partial least-squares structural equation modeling was performed using SmartPLS 3 software to test the proposed hypotheses statistically. A bootstrapping test was used to identify the mediating effects.

**Results:**

The main findings show that: (i) OC is the most powerful factor in promoting employees’ OVI (*β* = 0.26), while organizational culture (represented by the concept of internal market-oriented culture) and LAS showed significantly less and almost equal impact (*β* = 0.16 and *β* = 0.15, respectively). In total, OC, organizational culture and LAS explain 25% of the variance in the concept of OVI. (ii) LAS and organizational culture both significantly contribute to employees’ OC (*β* = 0.35 and *β* = 0.29, respectively) and in total explain nearly 40% (*R*^2^ = 0.38) of the variance in the concept of OC. (iii) The relationships between organizational culture, LAS, and OVI are mediated through OC, and (iv) LAS mediates the relationship between organizational culture and OVI, and that between organizational culture and OC.

**Conclusions:**

To promote hospital employees’ OVI effectively, hospital managers should focus particularly on their employees’ OC. Specifically, they should strengthen their employees’ OC through building a strong employee-focused organizational culture and ensuring that leaders practice LAS. This contributes to promoting hospital employees’ OVI.

**Supplementary Information:**

The online version contains supplementary material available at 10.1186/s12913-021-07430-z.

## Background

You may have personally experienced this! You are participating in a workshop, often located far away from the hospital where you are employed. You and your colleagues work from early morning until late evening. Finally, after 2–3 days of intensive collaborative work, a 10-page document is produced. When returning home, all participants are happy and proud of the final result. When you arrive at the office the next day, you file the document produced during the workshop away somewhere. Upon sitting down in your office chair, you reflect, “That was it?” and ask yourself, “Will the results of the workshop really have any impact on the employees in our hospital organization, and will it lead to a desired outcome such as a positive increase in their work performance?”

Wait! What is this story about? What was the goal of the workshop? You have probably already guessed. The correct answer is attending a strategy workshop to develop a new vision for your hospital organization.

This opening vignette illustrates and highlights two fundamental questions that arise regarding the potential power of developing a new vision for hospital organizations. These questions are most probably also relevant for other organizations. The first question relates to the real effect of organizational vision, such as whether it will have any impact on hospital employees’ work performance. The second question focuses on whether the hospital employees will internalize the organizational vision. Clearly, both questions are of substantial importance. However, it is reasonable to argue that the second question is more fundamental and critical in its content compared with the first because internalization to a large extent constitutes a necessary precondition or an initial step towards the actual manifestation of tangible effects stemming from organizational vision, which is the focus of the first question. Consequently, without any internalization by hospital employees, a vision of hospital organization will have only a limited or no effect on hospital employees’ work performance. In such a situation, the organizational vision for the hospital can be considered relatively useless [1]. Slåtten and Mehmetoglu stress the critical importance of implementing organizational vision among the organization members: “Implementation is fundamental for a firm’s success” [1].

Historically, health-care organizations (e.g., hospitals) have seldom focused on or concerned themselves with aspects of organizational vision [2]. However, this has now changed, and many health-care organizations (e.g., hospitals) consider it important to have a vision for their organization [3]. There are two possible explanations for this change and for the increased focus on the importance of studying organizational vision in health-care services. First, the competitive environment among health-care service providers has changed dramatically in recent years from being relatively static to being significantly more dynamic or even turbulent. Many countries have experienced a substantial growth in the number of both private and public hospitals, the privatization of many health-care service offerings, and the introduction of a continuous stream of new and innovative health-care technologies. New technology has in many cases saved costs, increased productivity, and improved service quality offered to patients. Consequently, some health-care organizations have gained a significant advantage over their competitors. Second, because of the easy availability of information (e.g., on the Internet), it is no longer a problem for “customers” to evaluate and compare the service quality of most health-care service organizations and identify the “best in the class” or the “most successful” ones, and therefore consider them to be the most attractive service providers. Clearly, these two factors have driven the dynamism and intensity among competitors in the health-care industry. However, as a way to cope and maneuver in these challenging times with a “rapid pace of change” [2] health-care service organizations (e.g., hospitals) have seen the value of developing a vision for their organization [3]. The basic idea of an organizational vision is for this to function as mental compass and guide decisions by the organization and individual members regarding its desired future state [3]. Thus, an organizational vision should ideally provide employees with an understandable and clear focus and convince each employee to participate actively to fulfill it through their work performance. Consequently, a basic criterion for an appropriate and “good” hospital organizational vision is that it should inspire, motivate, and encourage each hospital employee to act in line with the vision statement. Although very few studies have been undertaken, previous health-service research has identified a positive relationship between hospital employees’ level of implementation of the organizational vision and their service effort [3]. Consequently, because hospitals—like most health-care service organizations—can be described as a human resource-intensive industry, it is important to identify factors or constellations of factors that can promote the implementation of organizational vision [3].

For the reasons discussed above, the purpose of this study is to examine the factors that promote the implementation of organizational vision, which is referred to in this study as *organizational vision integration* (OVI). Specifically, the study takes the employees’ perspective on OVI. This contrast with most previous research, which has primarily focused on the leadership perspective. Previous research has discussed the importance of organizational vision for health-care service organizations in relationship to factors such as: the importance of having a clear vision [4], assessing the quality of the vision statement and (financial) performance [5], and changes in perception of the organizational vision over time [6]. However, these previous studies have lacked a clear and explicit focus on the factors that encourage health-care organization employees to apply the vision statement. According to Kohles et al., [7] employees have been “only rarely mentioned in the visioning process … often relegated to a largely passive role in vision implementation.” This lack of focus on employees in the previous research literature is surprising, because it is employees who “ultimately determine whether vision statements are ignored or accepted” [7]. In a similar vein, Slåtten et al. noted that “it becomes fundamentally important to take an employee perspective when studying the integration of organizational vision” [3]. By taking an employee perspective, this study is to our knowledge among the first, and pioneering, studies in the health-care services research literature on what factors promote OVI among health-care employees. To our knowledge, only one previous study (Slåtten et al. [3]), has been from the employee perspective when studying OVI. It identified and examined two different factors in the promotion of OVI: employees’ experience of psychological capital and perceived organizational attractiveness. Both factors were found to be positively associated with OVI, and together explained 30% of the variance in OVI. On this basis, the authors stated “there is considerable variance left in OVI to be explained” [3]. Moreover, future authors were urged to focus on three levels of promoting factors—(i) organizational culture, (ii) leadership styles, and (iii) employee attachment to the organization—and to examine how these factors individually and collectively promote OVI. The present study follows the suggestions of Slåtten et al. [3]. Consequently, it extends and deepens previous research on OVI and thus contributes to a relatively neglected and unexplored domain in the health-care services research literature.

This study is structured as follows. First, we present the theoretical framework of the study whereby each concept is defined and the associations between concepts are hypothesized. Second, we elaborate on the methodology and present the results of the statistical tests. Third, we discuss our findings and provide suggestions for future research. Finally, we provide some conclusions.

## Theoretical framework

**Fig. 1:**
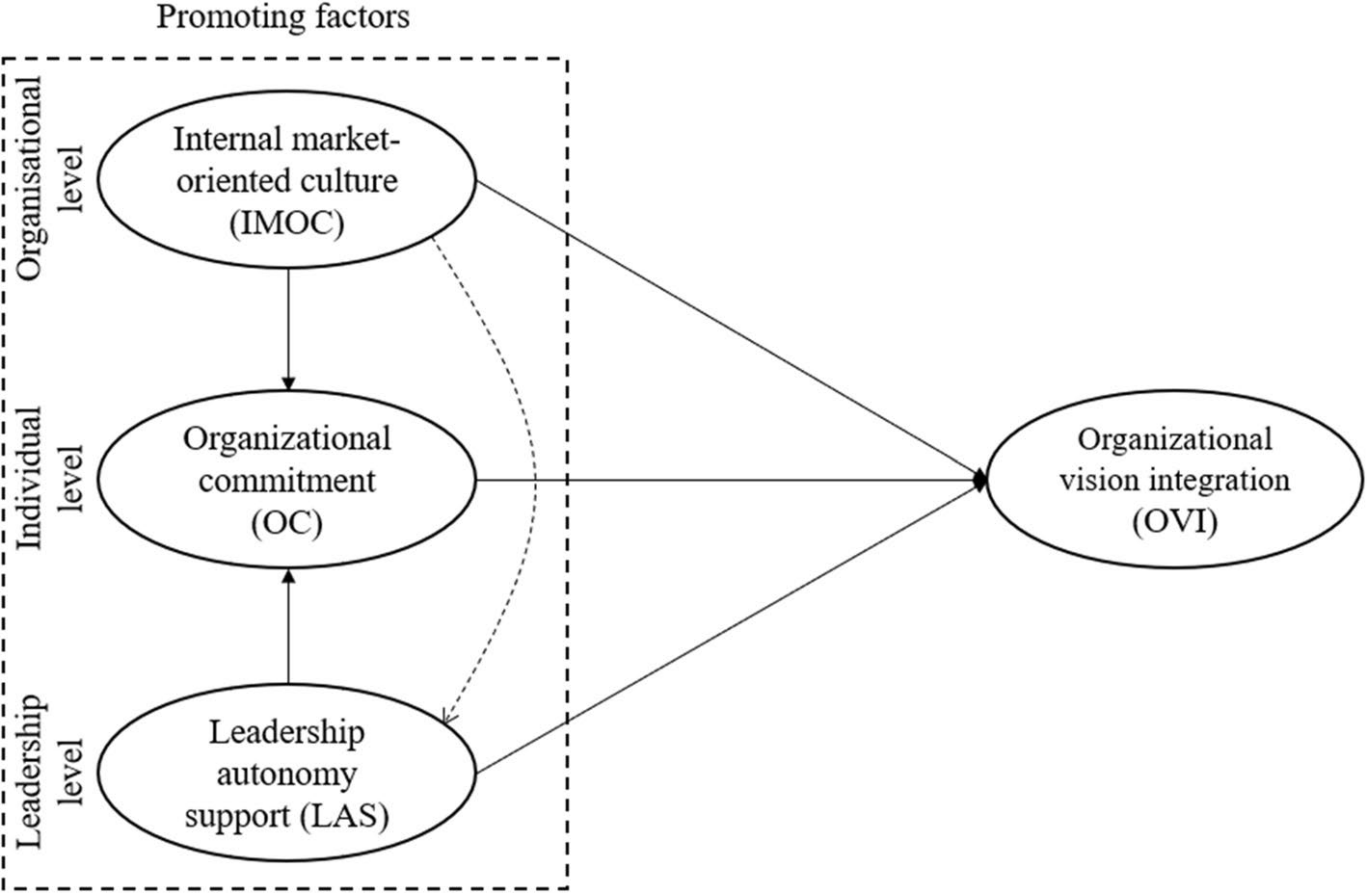
Conceptual model of factors that promote OVI

The following section describes the theoretical framework of the study. The section ends by summarizing the issues raised and presents the conceptual model (Fig. 1) to be examined in this study.

### Organizational vision integration (OVI)

As mentioned in the previous section, the aim and focus of this study is to consider organizational vision from an individual employee perspective, as shown in Fig. 1. In particular, it focuses on employees’ adoption and implementation of an organizational vision. These two elements (adoption and implementation) are both reflected in the concept of OVI, which is defined as “whether or not followers [employees] use the [organizational] vision as a guiding framework when making decisions and discretionary behaviors in their daily work roles” [7]. The adoption element is a cognitive aspect of OVI. It concerns capturing employees’ attention and knowledge, e.g., whether employees are familiar with and accordingly “know and understand the [organizational] vision” [7]. Although adoption (a cognitive aspect) is an important ingredient, it is insufficient on its own to explain the full meaning of OVI in this study. To capture it fully, the concept includes an implementation element, which is a behavioral aspect of OVI. Implementation concerns the employees’ conscious use of organizational “vision as a guiding framework in their particular jobs” [7]. Consequently, it is the combination of adoption (a cognitive aspect) and implementation (a behavioral aspect) that constitutes the concept of OVI. It is important to recognize that OVI does not focus exclusively on any specific level of the organization (e.g., the administrative level) nor is it directed at any specific job (e.g., frontline employees). In line with the conventional idea that organizational vision should be diffused throughout the organization, the concept of OVI is relevant to all members. Thus, if OVI is present among the organization’s individual members, it can be a powerful common and unifying guiding principle and a compass for all employees, regardless of their role.

The next section proposes three factors assumed to promote employees’ OVI. Each factor and reason for its inclusion will be discussed with its anticipated relationship to employees’ OVI.

### Factors that promote employees’ OVI

#### Organizational commitment (OC)

For organizations to become competitive and maintain their competitiveness, they need employees who are highly committed [8]. For this reason, their OC is assumed to be positively associated with OVI. As seen in Fig. 1, OC is an individual-level promoting factor. Specifically, OC concerns the “strength of investment in an organization by its employees” [9]. OC can be divided into three components representing distinctive features of employee investment in the organization: affective, continuance, and normative commitment [10, 11]. In this study, however, OC is represented by the affective component. Compared with the other two components (i.e., continuance and normative commitment), the affective component can be considered the most beneficial or “best” type of OC. In this study, employee OC, as an affective type of commitment, is defined as a psychological experience of a “positive emotional attachment to the organization” [12]. Here, OC represents a positive tie or bond between the employees and the organization. Consequently, OC in this study concerns the employees’ positive desire to be committed to their organization. The choice to limit the focus of this study to the affective component of OC is supported by previous research. According to Jafri [12], much research has “centered on the affective component” of OC [12].

In this study, it is supposed that employees’ OC is positively associated with their OVI. To achieve OVI is not necessarily an easy task for employees, as it can sometimes be challenging and demanding. The main reason for this claim is the true content and nature of an organizational vision statement. Kohles et al. observe that “vision statements may … represent an attempt to change employee behaviour” [7]. Describing and defining OVI in this study implicitly involves both potential cognitive changes (i.e., the adoption element of OVI) and behavioral changes (i.e., the implementation element). OVI is therefore a relatively demanding task for employees. Consequently, employees must have an inner desire, willingness, or motivation to engage in the OVI “work.” The content of a formal written employment contract, specifying and clarifying employees’ obligations and efforts in their jobs, does not necessarily normally include an explicit obligation to engage in OVI “work.” Thus, OVI can be described as voluntary employee behavior or what the literature labels “extra-role effort,” whereby employees decide, on a mainly individual basis, to engage in activities because they “want to do it” and not because “they have to.” There is thus a conscious choice to participate, stemming from source other than just a formal requirement. Accordingly, it is essential for another type of employment contract to exist, other than a formal, written one, to explain the motivational source and logical basis for employees’ OC and their level of OVI. Support for such a link between OC and OVI can be found in psychological contract theory. This is a central theory on the reasons for employee behavior in organizations [13]. As the name of the theory indicates, in contrast to a written contract, an inner mental psychological contract also exists that motivates or engages employees and has an impact on their level of “effort on behalf of the employer” [14]. It is reasonable to assume that similarly to employees’ OC, their psychological contracts reflect their level of positive attachment to their organization. Consequently, the nature and content of employees’ psychological contract and definition of OC in this study share some similarities. Because a psychological contract includes positive attachment to the organization, it to some extent explains why affectively committed employees participate in extra-role behavior such as the OVI work. Therefore, psychological contract theory supports the presumption that affectively committed employees (referring to OC) have the necessary motivational drive to engage or be involved in OVI. Support for a relationship between OC and OVI can also be found in previous research. According to Chen et al., “employees who have a strong identification with their organization [affective commitment] … are likely to make their best effort to benefit the organization” [9]. It is likely that such employees’ “best efforts” also entail OVI work. Chang et al. [15] describe affective commitment as an “employee’s emotional connection to the … goals of the organization”. Thus, OVI is an important goal for the organization to achieve and implicitly connects employees’ OVI positively with their OC. Consequently, this study of their affective component suggests that the more OC employees possess, the more it should promote their OVI. This leads to the following hypothesis.

- **Hypothesis 1:** OC is positively related to OVI.

### Internal market-oriented culture (IMOC)

Organizational culture has been suggested as a key factor in health-care organizations (e.g., hospitals) [16]. In this study, organizational culture is reflected in employees’ perceptions of IMOC. Specifically, as shown in Fig. 1, IMOC is an organizational-level factor that promotes employees’ OVI. In contrast to OC, based on job demands–resources (JD-R) theory [17], the concept of IMOC focuses on the significant motivational impact of employee perceptions of the organizational culture on their behavior [18]. This study focuses on the most observable component of an organizational culture, i.e., norms and behavior [18], which are reflected in the IMOC. IMOC emerges from the internal market orientation of the marketing domain [19]. The principle or “core idea of IMOC is to treat employees as customers” [20]. Parallel to the idea that it is important for managers of an organization to understand the needs and wants of its external customers and respond appropriately, the concept of IMOC reflects the importance “for managers to recognize the needs and wants of employees [or what can be described as internal customers] and … respond to these needs and wants … relevant to employees’ working conditions” [20]. IMOC concerns employees’ perceptions of the norm-based behavior of managers in the organization. It consists of three closely related parts: (i) internal market intelligence generation, (ii) internal intelligence dissemination, and (iii) response to internal intelligence [19]. Information is the common denominator, both within and across each of the three parts of IMOC. Specifically, internal market intelligence generation (part I) concerns the collection of information about needs and wants. Internal intelligence dissemination (part II) “concerns communication between employees and their managers, as well as between managers of different departments in the organization” [20]. Finally, response to internal intelligence (part III) concerns the managers’ specific action measures in response to the needs and wants identified in part I (internal market intelligence) and agreed upon in part II (internal intelligence dissemination). Naturally, for an organization to have a strong and powerful IMOC, all three IMOC parts must function well in with each other and be perceived as positive and beneficial by the organization’s employees.

Although few studies have been undertaken in a health-care setting, the previous research literature shows that IMOC is positively associated with a variety of aspects of employees’ work roles, such as level of engagement in work role, perceived attractiveness of their organization, and service quality provided to hospital patients [21]. Consequently, IMOC is an organizational factor that may motivate employees. In the present study, it is expected that IMOC can also promote OVI efforts. No previous research has examined the relationship between IMOC and OVI. However, the idea of a linkage between IMOC and OVI finds support from the JD-R theory [17]. This theory emphasizes that different types of resources in a work environment motivate employees to work [17]. As defined in this study, IMOC can be considered a supportive organizational resource that motivates employees’ efforts, such as their engagement in OVI. As Wan et al. observed, “a supportive work environment [in this study, IMOC] … fosters employees’ willingness to dedicate their effort and abilities to job tasks” [22]. Previous research has revealed that employee perceptions of organizational culture have a major impact on both their attitudes and behavior [23, 24]. Uniquely, in our study, it is assumed that IMOC, as a type of organizational culture, pervades “all aspects of organizational life” [25], including employees’ OVI efforts. Consequently, when employees perceive the organization’s IMOC to be favorable, it should be a motivational factor for OVI. For this reason, the following hypothesis is proposed.

- **Hypothesis 2:** IMOC is positively related to OVI.

It is also assumed that IMOC can indirectly promote OVI when OC mediates the relationship. OC is defined as employees’ “positive emotional attachment to the organization” [12]. The research literature has shown that employees’ emotions are always evoked by a specific factor or factors [26]. Consequently, there must be a reason why employees are positively emotionally attached to the organization (i.e., OC). In this study, the cause of OC is assumed to be IMOC. It is important to keep in mind that IMOC focuses “on more tangible or visible aspects of organizational culture that … hospital employees experience or observe daily” [20]. Because of the relatively observable nature of IMOC, there are good reasons to expect it to have a direct impact on employees’ OC. Although no study in the health-care services research literature has examined the relationship between IMOC and OC, previous research into the association between organizational culture and employees’ OC provides some support [27]. Naturally, there are variations in employee perceptions of their organizational IMOC, ranging from highly positive to highly negative. However, when IMOC is perceived positively, it should strengthen the employees’ OC (emotional attachment). Furthermore, their OC then increases because of their more favorable perceptions of IMOC, which should also positively reinforce their OVI. According to Lages and Piercy, affectively committed employees are more motivated and willing to “go beyond the job specification” [28] to make an extra effort and contribute positively to organizational development. Accordingly, IMOC can “fuel” employees’ OC, encouraging them to make extra-role efforts, manifested in their OVI. This reasoning implies that OC has a mediating role between IMOC and OVI.

The above discussion can be summarized by the following two hypotheses.

- **Hypothesis 3:** IMOC is positively related to OC.
- **Hypothesis 4:** The relationship between IMOC and OVI is mediated by OC.

### Leadership autonomy support (LAS)

Leadership in organizations (e.g., hospitals) is clearly a primary influence on employees in organizations [29]. As shown in Fig. 1, compared with OC (an individual-level promoting factor) and IMOC (an organizational-level promoting factor), LAS represents a leadership-level promoting factor for employees’ OVI. Because of the central role of leaders in the organization, in addition to their formal authority, leaders undoubtedly constitute a powerful influence on employees [29]. For many employees, leadership behavior is largely seen as a principal factor in their motivation and optimal performance at their workplace [30, 31]. In this study, these positive leadership aspects are embraced in the concept of LAS, which is a “leadership style that is thought to nurture the inner motivational resources of employees” [30]. LAS focuses on the interpersonal relationship between employees and their leaders and how it is perceived by employees. LAS is manifested in interpersonal relationships when employees perceive their leader as a person who provides “a meaningful rationale for doing the tasks, emphasize[s] choice rather than control, and acknowledge[s] employees’ feelings and perspectives’” [32]. LAS is about the capability of leaders to inspire and encourage their employees in a positive manner to think and act autonomously. Implicitly, LAS means that leaders do not exercise controlling behavior over employees. Consequently, LAS stimulates employees to use their freedom or autonomy to take initiatives and make choices and decisions that benefit their work.

It is feasible to suspect that such initiatives and choices facilitated by LAS are related to the employees’ OVI. As noted in the previous discussion, OVI is about the employees’ use of organizational vision as a “guiding framework when making decisions and discretionary behaviors in their daily work roles” [7]. As OVI includes both a cognitive aspect (adoption of vision) as well as a behavioral aspect (implementation of vision), it is a relatively demanding and complex task. In addition, OVI is an extra-role effort that employees make because “they want to,” not because “they have to.” To achieve OVI, employees need a “reservoir” that includes both autonomy and motivation (i.e., autonomous motivation) to initiate the necessary cognitive and behavioral changes embraced by OVI. In the literature, LAS is closely related to employees’ autonomous motivation [32]. Consequently, LAS is considered to provide employees with the necessary ingredients for generating and nurturing their OVI. The proposed linkage between LAS and OVI also finds support from the job demands–resources (JD-R) theory [17]. As mentioned above, this theory focuses on different types of resources in a work environment motivating employees to perform their work tasks [17]. Clearly, LAS is a relevant type of resource in the work environment that has an impact on their motivation to engage not only in predefined role tasks but also in extra-role behavior. Similar to the way a supportive work environment can motivate employees to dedicate the necessary effort to their work [33], LAS is expected to promote work effort, manifested in their OVI. Thus, the following hypothesis is proposed.

- **Hypothesis 5:** LAS is positively related to OVI.

As shown in Fig. 1, LAS may promote OVI in an alternative manner. In particular, employees’ perceptions of their employer, as represented by their OC, mediate their perceptions of LAS and their OVI. Previous research supports the view that employees’ sense of autonomy, reflected in their perceptions of control and decision-making authority in their work role, is positively related to their OC [34]. Therefore, in situations where employees perceive or find the LAS practice to be positive, it should strengthen employees’ OC. Prior studies have shown that LAS and OC (defined as affective commitment) are positively related [15]. Consequently, when employees’ OC (employees’ affective commitment) increases as a result of their positive perceptions of LAS, it should also strengthen or reinforce their motivation to do what is in the best interests of organizational development. Therefore, this effect of strengthening employees’ OC because of LAS is related to their willingness to undertake the extra-role effort or “work” regarding OVI. Previous research found that OC (defined as employees’ affective commitment) positively promotes beneficial job-related outcomes [35] and employees’ efforts to “go beyond job specifications” [28]. This reasoning suggests that OC plays a mediating role between LAS and OVI. Based on this reasoning, two further hypotheses are proposed.

- **Hypothesis 6:** LAS is positively related to OC.
- **Hypothesis 7:** The relationship between LAS and OVI is mediated by OC.

Leadership is “among the most dominant factors” influencing employees [20]. However, the application of a leadership style is always embedded within a larger organizational context. Thus, it is expected that the leadership style in this study, represented by LAS, operates in a symbiotic relationship where LAS is affected by and affects other relevant factors within the organizational context. This study attempts to reveal the role of LAS in an organizational context in relation to IMOC, OC, and OVI. Notably, as shown in Fig. 1, this study explores whether LAS acts as a mediating factor between IMOC (at the organizational level) and employees’ OC and OVI (at the individual level). To the best of our knowledge, few studies within the health-care services research literature have examined these relationships.

A basic or fundamental premise for the suggestion of LAS as a mediating factor is that it is changeable. LAS is not a static construct; it is dynamic but controllable and manageable. Thus, IMOC can regulate LAS. IMOC concerns employees’ perceptions of their organization and in particular reflects their perceptions of how well “managers recognize the needs and wants of employees and … respond to these needs and wants … relevant to employees’ working conditions” [20]. Given its nature and content, IMOC can be expected to impact employees’ experience of supportiveness in terms of autonomy from their leaders (i.e., LAS). With its strong focus on understanding employees’ needs and wants, IMOC can be described as a type of supportive organizational culture. Consequently, it is reasonable to presume a close relationship between IMOC and LAS.

Most employees do not prefer leaders who focus on control. In contrast, employees appreciate leaders who give them freedom and support their autonomy in their work role. Consequently, in an organization with a strong and positive IMOC, leaders naturally respond to employees’ needs and desire for autonomy. Therefore, IMOC in organizations provides leaders with behavioral norms and serves as a mental guide or inner map for LAS behavior in their organization. This impact of organizational culture on leadership behavior is consistent with findings in the research literature. For example, Banaszak-Holl et al. stress the importance of organizational culture and describe it as a “key mechanism by which top management integrate managerial actions” [25]. Studies have positively correlated organizational culture and leadership behavior [36, 37]. Employees’ perceptions of IMOC in their organization vary from strongly negative to strongly positive; however, this study takes a positive perspective on the impact of IMOC. In particular, because of the dynamic nature of LAS it is assumed that IMOC can manage LAS positively. Consequently, when LAS increases because of employees’ more favorable perceptions of IMOC, this should also lead to employees being more affectively committed to their organization (i.e., OC). Furthermore, an increase in LAS because of IMOC should simultaneously stimulate and motivate employees to dedicate more of their inner motivation, energy, and effort to working for the benefit of their organization, including involving themselves in extra-role work efforts related to OVI. Consequently, it is expected that employees’ perceptions of LAS are a mediating factor between IMOC and employees’ OC and OVI. Thus, the mediating role of LAS constitutes the two final hypotheses proposed in this study.

- **Hypothesis 8:** LAS mediates the relationship between IMOC and OC.
- **Hypothesis 9:** LAS mediates the relationship between IMOC and OVI.

Based on all aspects addressed in the above discussion, three types of promoting factors of OVI are included in this study. As shown in Fig. 1, these are: (i) internal market-oriented culture (IMOC), (ii) organizational commitment (OC), and (iii) leadership autonomy support (LAS).

IMOC, OC, and LAS are shown in the dotted square in Fig. 1 and represent the three idiosyncratic levels of promoting factors: IMOC represents the *organizational* level, OC represents the *individual* level, and LAS represents the *leadership* level. Although IMOC, OC, and LAS are distinctive, they are (as also shown in Fig. 1) suggested to be interrelated in promoting OVI. As shown in Fig. 1, OC, IMOC, and LAS are suggested to be directly related to employees’ OVI. In addition, it is assumed that the relationships between IMOC, LAS, and OVI are mediated through OC and the relationships between IMOC, OC, and OVI are mediated through LAS. All nine hypotheses suggested in this study are summarized in Table 1.

**Table 1:** Hypotheses used in this study

Hypothesis	Hypothesized relationships
H1	OC is positively related to OVI
H2	IMOC is positively related to OVI
H3	IMOC is positively related to OC
H4	The relationship between IMOC and OVI is mediated by OC
H5	LAS is positively related to OVI
H6	LAS is positively related to OC
H7	The relationship between LAS and OVI is mediated by OC
H8	LAS mediates the relationship between IMOC and OC
H9	LAS mediates the relationship between IMOC and OVI

Note: *IMOC* Internal market-oriented culture, *LAS* Leadership autonomy support, *OC* Organizational commitment, *OVI* Organizational vision integration

## Methods

This study aimed to examine the factors that promote OVI among hospital employees. The director of research (DOR) of a hospital organization, who is also a member of the largest health expert communities situated in the inland counties of Norway, was contacted. The hospital organization has more than 10,000 employees. After accepting our invitation, the DOR disseminated all the information about the survey to the division managers, staff units, and department managers at the hospital. A cross-sectional survey was used to collect employee data, although cross-sectional studies have issues with common method variance (CMV) [38]. With the help of several selected hospital employees and three academic experts translations from English to Norwegian were performed to verify the translation of the adapted items. To control for the CMV issues, we followed the guidelines of Podsakoff et al. [39]. For example, we constructed a survey using established constructs. We ensured that the claims were simplified, specific, and concise, and restructured questions relating to more than one possibility into simpler, more focused questions. Moreover, we avoided vague concepts in the survey. Thereafter, several pretests were performed to ensure the quality of the items included in the survey. In addition, the survey was discussed and pretested on two academic professionals to ensure its overall quality. Prior to distributing the survey, the final questionnaire was sent to the Norwegian Centre for Research Data (NSD) for approval of the study, and to ensure that the study followed the proposed national research ethics guidelines. The DOR also agreed to assist in the distribution of the survey. First, the survey was distributed to division managers and department managers, who forwarded it to their employees. This survey was part of a larger project initiated in February 2020. Owing to the project’s restricted time frame, various data collection methods such as longitudinal or panel data were ruled out. In addition, based on previous studies of OVI [e.g., 7] and our goals, and in agreement with the DOR, an online survey was chosen as the optimal sampling technique to gather data on the promoting factors of OVI. To ensure participant anonymity and avoid nonresponse bias, the online survey was distributed through a platform called *Nettskjema,* which offers full anonymity through automatic deletion of IP addresses upon completion of the online survey. Although there were some minor differences between divisions and departments, it is important to note that this study focused on employees’ perceptions of their organizational vision and not on the divisional or departmental differences. In total, 2000 hospital employees across seven different departments were invited to participate. Through convenience sampling, a total of 1008 hospital employees returned completed questionnaires: a response rate of 50.4%. The respondents were asked to specify whether they held leadership roles or responsibilities. Upon receiving the data, we excluded respondents who reported leadership roles or responsibilities. Thus, our study only included employees, nurses, and doctors without leadership responsibilities (including those categorized as “others”). Therefore, the distribution of the survey helped ensure that all employees included in the analysis were in similar categories and levels. This is consistent with Carlucci’s [40] empirical examination of ways to foster innovative employee behavior in health-care organizations. Harmonious, we surveyed IMOC and LAS from the employees’ perceptions of their organization and their leaders. Table 2 summarizes the personal characteristics of the study sample.

**Table 2:** Personal characteristics of the study sample (*N* = 1008)

	%
Sex	
Female	73.0
Male	27.0
Staff role	
Nurse	33.0
Doctor	8.7
Others (admin staff, other health professionals, etc.)	58.3
Duration of employment	
< 5 years	26.9
6–10 years	18.0
11–20 years	30.3
> 20 years	24.8
Part-time or full-time job	
Part-time	22.5
Full-time	77.5
Age	
< 45 years	37.3
46–55 years	32.2
> 55 years	30.5

### Instruments

The proposed conceptual model (Fig. 1) includes four constructs: OVI, OC, IMOC, and LAS. Although all claims for each construct were based on previous research, adaptation was necessary to make them more relevant and appropriate for the health-care setting. A seven-point Likert scale ranging from (1) strongly disagree to (7) strongly agree was used for all items. The items for the concept of OVI were adopted from Liu [41] and Slåtten and Mehmetoglu [42]. The items representing the concept of OC were adopted from Allen and Meyer [10]. Those used for LAS were adopted from Amundsen [43]. IMOC was measured using items from Slåtten et al. [21]. As mentioned above, the items used in this study are part of a larger project focusing on various aspects of employee relations in hospital organizations. Table 3 shows the items for the four concepts in this study.

**Table 3:** Constructs (i.e., IMOC, LAS, OC, and OVI) and claims used in the study

Construct	Claims label	Claims
IMOC	IMOC1	Employees have the opportunity to discuss their needs with management.
	IMOC2	Training is seen in the context of individual needs.
	IMOC3	Management spends time talking to their employees when needed.
	IMOC4	Management wants employees to enjoy their work.
	IMOC5	Management shows a sincere interest in any problems faced by employees.
	IMOC6	Management understands that personal problems may affect my performance.
	IMOC7	The division’s policies help meet employees’ individual needs.
	IMOC8	Management meets regularly to discuss issues related to employees’ challenges.
LAS	LAS1	My leader gives me authority over issues within my area.
	LAS2	My leader listens to me.
	LAS3	My leader encourages me to take initiative.
	LAS4	My leader is concerned that my work is goal-oriented.
	LAS5	My leader instils motivation.
OC	OC1	I am proud to tell others that I work here.
	OC2	I feel I belong to this organization.
	OC3	I feel personally attached to my organization.
	OC4	I envision a career at this organization.
	OC5	I want to continue my career here.
OVI	OVI1	The management has informed me about the company’s vision and aim.
	OVI2	I am familiar with the organization’s vision and aim.
	OVI3	I am conscious of doing my job in line with the company’s vision and aim.

Note: *IMOC* Internal Market-Oriented Culture, *LAS* Leadership autonomy support, *OC* Organizational commitment, *OVI* Organizational vision integration

### Data analysis

Using SmartPLS 3 software, partial least-squares structural equation modeling (PLS-SEM) was used to test the hypotheses [44]. The first step in evaluating PLS-SEM results involved examination of the measurement model, which consisted only of reflective measures. The second step was to assess the structural model. Based on the PLS-SEM results, the mediating effects were also estimated and analyzed using the bootstrapping test of Zhao et al. [45].

## Results

### Measurement model

To assess the reflective measurement model, we examined convergent validity, internal consistency reliability, and discriminant validity. Convergent validity is the extent to which a variable is positively correlated with alternative variables used to measure the same construct. The construct can be judged by internal consistency, assessed by the magnitudes of the intercorrelations of the observed variables. Discriminant validity is the extent to which a construct is distinct from other constructs, assessed with the heterotrait–monotrait (HTMT) ratio of correlations between constructs. The evaluations of the results for convergent validity, internal consistency, and discriminant validity set out in Table 4 all satisfy the “rule of thumb” criteria of Hair et al. [38, 46, 47]; this supports the view that we have a reliable and valid measurement model.

**Table 4:** Results of the measurement model for the IMOC, LAS, OC, and OVI constructs

		Convergent validity		Internal consistency reliability		Discriminant validity
Construct	Claims label	Indicator reliability	AVE	Composite reliability	Cronbach’s alpha	HTMT criterion
Rule of thumb		Loading > 0.7	> 0.5	0.7–0.95	0.7–0.95	HTMT interval does not include 1
IMOC	IMOC1	0.84	0.73	0.95	0.95	Yes
	IMOC2	0.76				
	IMOC3	0.89				
	IMOC4	0.86				
	IMOC5	0.90				
	IMOC6	0.84				
	IMOC7	0.82				
	IMOC8	0.90				
LAS	LAS1	0.83	0.80	0.95	0.94	Yes
	LAS2	0.92				
	LAS3	0.93				
	LAS4	0.85				
	LAS5	0.92				
OC	OC1	0.86	0.72	0.93	0.90	Yes
	OC2	0.88				
	OC3	0.84				
	OC4	0.84				
	OC5	0.83				
OVI	OVI1	0.90	0.81	0.93	0.88	Yes
	OVI2	0.92				
	OVI3	0.87				

Note: *AVE* Average variance extracted, *HTMT* Heterotrait–monotrait ratio of correlations, *IMOC* Internal market-oriented culture, *LAS* Leadership autonomy support, *OC* Organizational commitment, *OVI* Organizational vision integration

### Structural model

Before we assessed the structural model, we examined multicollinearity between the latent constructs using the variance inflation factor (VIF). VIF values of > 5 indicate multicollinearity issues [47]. All VIF values were < 4, indicating no multicollinearity problems.

**Fig. 2:**
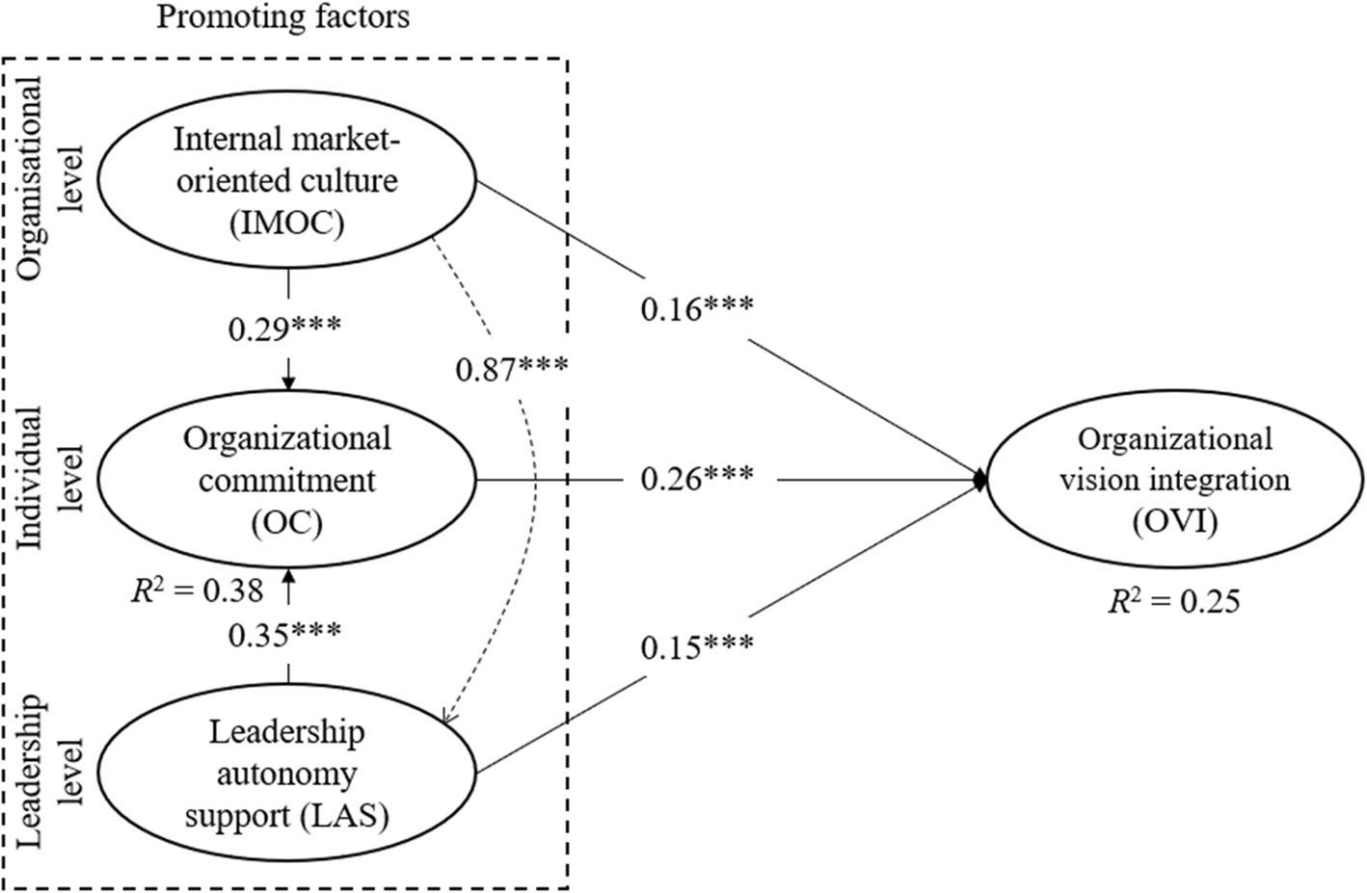
Results from the structural model for factors that trigger OVI. Standardized coefficients (***p < 0.01)

The direct effects in the structural model are shown in Fig. 2. For the endogenous constructs, we examined the in-sample predictive power of the model using *R*^2^. The *R*^2^ values for OC and OVI were 0.38 and 0.25, respectively. Based on the “rule of thumb” [46, 47], these *R*^2^ values were considered moderate. All the standardized direct-path coefficients were statistically significant at the 1% significance level. The path coefficient between IMOC and LAS was highest at 0.87, and the second-highest path coefficient of 0.35 was between LAS and OC. The relationship between OC and OVI was positive (*β* = 0.26), supporting H1. H2 and H3 were also supported, because the relationships between IMOC and OVI and between IMOC and OC were positive (*β* = 0.16 and *β* = 0.29, respectively). LAS was positively related to OVI (*β* = 0.15), supporting H5. Finally, there was a positive relationship between LAS and OC (*β* = 0.38), supporting H6.

To test the mediating effect in the models, we applied a bootstrapping test proposed by Zhao et al. [45] to assess whether the estimated direct and indirect effects were statistically significant. Depending on their significance, we could then determine which if any of the following effects exist: direct effects only, without mediation; no-effect nonmediation; complementary mediation; competitive mediation (direct and indirect effects are significant, but in the opposite direction); or indirect-only mediation.

The results for the various hypotheses are presented in Table 5.

**Table 5:** Test of mediating effects^c^ for LAS and OC

Hypothesis	Effect^a^	Mediating factor	Direct effect^a^	Indirect effect^a^	Mediating effect^b^
H4	IMOC → OVI	OC	0.157^***^	0.154^***^	Complementary
H7	LAS → OVI	OC	0.153^***^	0.091^***^	Complementary
H8	IMOC → OC	LAS	0.288^***^	0.075^***^	Complementary
H9	IMOC → OVI	LAS	0.157^***^	0.214^***^	Complementary

^a^ ** *p* < 0.05, *** *p* < 0.01 are significance levels

^b^ The effect between IMOC and OVI (H4) was influenced twice by the mediating factor OC, and we have a double-mediation analysis [34]. The same applied for the effect between IMOC and OVI and the mediating factor LAS (H9). The total indirect effect is then the sum of the specific indirect effects

^c^ Mediation by bootstrapping method [33]

Table 5 shows that OC had significantly positive direct and indirect effects in addition to a complementary mediating effect on the relationship between IMOC and OVI, supporting H4. OC was also found to complementarily mediate the relationship between LAS and OVI (with both significantly positive direct and indirect effects), supporting H7. There was a significantly positive direct effect between IMOC and OC, and a significantly positive indirect effect of LAS between IMOC and OC, implying a complementary mediating effect, supporting H8. LAS was also shown to have a significantly positive indirect effect and a complementary mediating effect between IMOC and OVI, supporting H9.

## Discussion

This study contributes to our understanding of organizational vision by examining the premises or motivating factors that may stimulate and motivate hospital employees to put the organizational vision into (everyday) practice through their actions and attitudes. We consider our study as being among the pioneering works in the health-care services research literature on OVI. Specifically, it offers three contributions. First, it studies organizational vision from an employee perspective, which has been relatively neglected in previous research [7]. Second, it reveals how factors at different levels (i.e., leadership, individual, and organizational levels) can promote employees’ OVI. Third, it explores the underlying pattern of relationships whereby factors at different levels interact and function in together to promote employees’ OVI. Given the focus of this study, it responds to a call for more research on employees’ OVI in health-care organizations [3, 48].

According to Foster and Akdere, “individual perception of vision is important because it is the individuals within the organization who actually put the vision into action” [49]. In line with this, the main concept in this study, OVI, refers to employees’ use of organizational vision “as a guiding framework when making decisions and discretionary behaviors in their daily work roles” [7].

A comparison of the direct impact of the three levels of factors that promote OVI reveals that the impact of OC was clearly dominant. The impact of OC was almost double that of the individual impacts of IMOC and LAS on OVI. Clearly, this major impact of OC has practical implications for hospital managers, who should place particular focus on employees’ perceptions of OC as a strategy to stimulate and manage employees’ OVI. OC, as a positive affective component, in this study refers to employees’ “emotional attachment to the organization” [12]. Consequently, when hospital employees have good feelings about their organization and are emotionally connected to it, they are motivated and more willing to devote the necessary time, energy, and personal effort to extra-role behavior and OVI.

To the authors’ knowledge, this is the first study that examines the relationship between employees’ OC and OVI in the health-care services context. However, the findings in this study are supported by two studies in other organizational contexts. Despite the different empirical contexts, they are both relevant as they share common features with the core ideas and concepts examined in our study. These are the studies by Chai et al. [8] and Dvir et al. [50]. In the first study by Chai et al., [8] the authors studied 455 work teams in the food services industry in South Korea. Specifically, Chai et al. [8] examined the link between employees’ OC and a concept they referred to as a “shared vision” [8]. OC was defined by Chai et al. [8] as affective commitment and in that respect, there are similarities with our study. The concept of a “shared vision” was defined as “the collective understanding of an organization’s vision, mission and core values among members of a group” [8]. Although Chai et al. [8] studied shared vision as a “collective understanding” rather than at the individual level, as we have done in this study, they found empirical support for a link between employees’ OC and shared organizational vision. The second study that supports our findings is by Dvir et al. [50]. As in our study, Dvir et al., [50] defined employees’ OC as affective commitment and linked it to what they termed “vision assimilation” among organizational members. Vision assimilation was “employees’ perceptions of vision clarity, sharedness, and appropriateness” [50]. Based on data collected from 183 employees employed in six Israeli high-technology firms, Dvir et al. [50] found support for a link between OC and vision assimilation among employees in the organization.

The abovementioned study of Dvir et al. [50] is also interesting for another reason. It highlights the importance of specifically developing the affective component of OC among members of the organization. Interestingly, the authors examined the impact of two different or diametrically opposed types of OC on employees’ vision assimilation. In addition to including the impact of OC as an affective commitment, Dvir et al. [50] also examined it as a cognitive commitment. In their study, OC as a cognitive type of commitment was described as more rational in its focus and it reflected a “calculative dimension [of OC] of the linkage between employees and their organization.” Consequently, and simply stated, Dvir et al. [50] tested the impact of employees’ “warm” OC (affective commitment) as well their “cold” OC (cognitive commitment) on their vision assimilation. Interestingly, the authors’ findings revealed that the warm OC (affective commitment) was positively related to vision assimilation, while the cold OC (cognitive commitment) was not. The findings of Dvir et al. [50] and this study clearly highlight the value of warm OC among organizational members. A practical implication based on the positive relationship between OC and OVI is the importance of hospital managers focusing on strengthening their employees’ warm OC (i.e., their affective commitment) because it is key to promoting OVI. This recommendation to focus on employees’ affective OC is consistent with Ryu’s [51]observation that “in prior research, affective commitment has shown to have the strongest and most favorable relations with organization-relevant and employee-relevant outcomes”. Consequently, hospital managers should note that the warmer the OC among hospital employees is, the more it can promote OVI.

In previous research studies, defining employees’ OC in the same manner as we have done in this study (referring to OC as an affective component), this type of OC is proposed to be closely and positively associated with a value congruence and person–organization fit [51]. Person–organization fit is about the “congruence between norms and values of organizations and the values of persons” [52]. This value congruence could emerge from employees’ perceptions of organizational goals, climatic conditions, cultural aspects, or other organizational aspects that they appreciate and consider of high personal value. Consequently, because OC is defined in this study as employees’ “positive emotional attachment to the organization” [12], it is plausible that their value congruence with their organization is embedded in and reflected in their OC. This may be an underlying reason why OC was found to be a stronger factor in promoting employees’ OVI than the impact of IMOC and LAS in this study. Ryu supports this reasoning, stating: “when employees perceive higher value congruence with their organization, they are more likely to feel integrally involved with the vison of the organization” [51]. The findings from this study indicate that employees’ OVI is predominantly promoted through their OC. Put another way, OVI is driven primarily by an *individual*-level promoting factor. Consequently, employees’ OC can be characterized as the main source of OVI among organizational members.

Although OC was undoubtedly the strongest factor directly promoting OVI (*β* = 0.26), this is not to say that *organizational*-level (IMOC) or *leadership*-level (LAS) factors are unimportant or uninteresting. Although they show less impact than OC, both IMOC and LAS were found to have a direct impact on OVI (*β* = 0.16 and, *β* = 0.15, respectively). Collectively, the three promoting factors explained 25% of the variance of OVI. Consequently, hospital managers should be aware that their employees’ perceptions of the IMOC and LAS in their hospital organization affects their willingness to work on their OVI. However, the role and value of IMOC and LAS become highly visible, especially given their indirect impact on employees’ OVI. Consequently, the findings of this study indicate that IMOC and LAS have direct impacts on OVI and that both facilitate OVI, which works mainly through employees’ OC.

OC can be described as a psychological state [53] and not a fixed personal trait. Consequently, because OC is a psychological state, it is dynamic and may vary as time passes depending on the influence or impact of environmental or contextually relevant factors, such as leadership and organizational factors. This implies that employees’ OC can be managed and “controlled” by the organization. The findings from this study suggest that both IMOC and LAS are highly influential and capable of managing OC (*β* = 0.29 and, *β* = 0.35, respectively). IMOC and LAS were found to explain almost 40% of the variance in OC (*R*^2^ = 0.38), which can be considered substantial explanatory power. Clearly, both IMOC and LAS are essential “fuel” ingredients that hospital managers can use to “warm up” their employees’ OC (i.e., affective commitment). Thus, we find that IMOC and LAS can be used intentionally by hospital managers to improve employees’ OC in hospital organizations.

However, the impacts of IMOC and LAS are not limited to their direct effect on OC. Based on the sophisticated statistical tests for mediation suggested by Zhao et al. [45], the IMOC and LAS in all mediation tests showed what Zhao et al. [45] describe as “complementary mediation.” According to Zhao et al. [45], complementary mediation indicates the presence of both direct and mediating effects where they “exist and point in the same direction.” Consequently, complementary mediation implies that IMOC and LAS, in addition to their direct impact on OC, can simultaneously promote OVI indirectly through OC. Furthermore, in the literature, organizational culture is suggested as pervading “all aspects of organizational life” [25]. The use of IMOC in this study to capture organizational culture supports the all-encompassing impact of culture. In addition to its multiple direct and indirect impacts described above in relation to OVI and OC, the significant direct impact of IMOC strengthens LAS in the hospital organization (*β* = 0.87) as well as the potential for IMOC to have an indirect impact on OC and OVI through LAS. Based on the mediation test of Zhao et al. [45], this indirect impact was also found to constitute “complementary mediation.” Consequently, a practical implication of this result is the importance for hospital managers to continuously track employees’ perceptions of the IMOC and LAS. IMOC and LAS, individually, collectively, and together with OC, directly promote OVI while the former two can—in multiple ways—also indirectly promote OVI through the employees’ OC.

### Limitations and future research

Studies on the concept of organizational vision in the health-care services literature are relatively scarce; therefore, there is an urgent need for more substantial research into this important research domain. This leads us to identify four limitations of this study that offer avenues for future research.

First, although we followed the recommendations and guidelines of cross-sectional studies [39], the study design has limitations. For example, this study focused on a single hospital organization in Norway. Although it was large, there are limitations connected to studying a single organization in terms of generalizability and robustness. Additional limitations, as mentioned above, are found in the study’s cross-sectional design, which risks self-selection bias and impedes inference of causality. Therefore, it is advised that future research studies gather data in distinctive time periods, panel data, or longitudinal data, while exploring the potential of a causal relationship among the studied factors. Collecting data from various contexts to minimize method bias is also a limitation of this study that offers future research opportunities. In addition, in cross-sectional studies, the CMV issue is present. Although procedures were followed to minimize such issues, as mentioned above, future research can obtain measures of predictor and criterion variables from a variety of sources.

Second, as shown in this study, the individual-level OC factor was the strongest promoter of OVI. Consequently, based on this finding, future research should go into greater depth to explore other potential individual-level factors and their relationship with OVI. As indicated in the previous discussion, one potential reason for the strong impacts of OC and OVI could be that higher employee OC was also associated with greater value congruence between employees and their organization. According to Ryu, [51] those employees “who perceive higher value congruence with their organizations are more likely to accept organizational vision”. Future research should strive to identify those common values or constellations of values that employees appreciate and perceive as good, and they are congruent with their organizations. Collecting data about employees’ value congruence would indicate whether this individual-level promoting factor in addition to OC can increase the explained variance in employees’ OVI.

Third, Kantabutra and Avery commented that “in today’s corporate world, we can observe that vision statements appear with a wide variety of characteristics” [54]. Hospital organizations are no exception; therefore, there is a need to understand what a “powerful” vision looks like [54]. To do this, as this study does, one should take an employee perspective to reveal what Zaccaro and Banks call “self-identification with vision” [55]. The impact of a vision statement on OVI could also be studied simultaneously with employees’ value congruence, OC, and perceived IMOC and LAS.

Fourth, although the literature describes several aspects that should be considered and those that a vision “must have,” e.g., clarity, future orientation, challenge, conciseness, ability to inspire [56], and so forth, we do not know the extent to which the fulfillment of either individual aspects or constellations of these aspects of an organizational vision statement can induce OVI among hospital employees. Identifying such aspects could have practical implications for hospital leaders seeking to increase the effectiveness and efficiency of their employees’ OVI.

## Conclusions

This study contributes to our understanding of promoting OVI in employees of hospital organizations. The study reveals that to promote OVI successfully, hospital managers should focus on their employees’ OC and strengthen it by building a constructive IMOC while having leaders who practice LAS. This contributes directly and indirectly to promoting employees’ OVI in hospital organizations.

## Supplementary Information

**Additional file 1.**

## Data Availability

The datasets used and/or analyzed during the current study are available from the corresponding author on reasonable request.

## Abbreviations

AVE: Average variance extracted
DOR: Director of Research
HTMT: Heterotrait–monotrait
IMOC: Internal market-oriented culture
LAS: Leadership autonomy support
NSD: Norwegian Centre for Research Data
OC: Organizational commitment
OVI: Organizational vision integration
PLS-SEM: Partial least-squares structural equation modeling
CMV: Common method variance
